# Protective factors against the emotional impact of the pandemic in adults with autism spectrum disorders (ASD) and intellectual disability (ID)

**DOI:** 10.1038/s41598-024-55049-x

**Published:** 2024-02-22

**Authors:** Marina Jodra, Domingo García-Villamisar

**Affiliations:** 1 Department of Personality, Evaluation and Clinical Psychology, Universidad Complutense de Madrid, Rector Royo Villanova s/n, Ciudad Universitaria, 28040 Madrid, Spain; 2 Asociación Nuevo Horizonte, Las Rozas, Madrid, Spain

**Keywords:** Intellectual disability, Adults, Protective factors, Autism spectrum disorders, Pandemic, Emotional impact, Disability, Human behaviour

## Abstract

The pandemic has had very negative effects on the mental health of the population, especially in people with autism spectrum disorders (ASD) and intellectual disability (ID). We analyzed whether social communication, quality of life, and anxiety explain changes in the emotional impact of the pandemic in 60 adults with ASD and ID. Correlations between the study variables were analyzed and subsequently a multiple regression analysis was performed. The results show that communication writing, leisure and well-being index, explain 31% of the dependent variable. The well-being index (PWI) contributes significantly to improving the fit of the model, as indicated by β value. The remaining variables, communication writing and leisure socialization, do not contributed significantly to improving the fit of the model. Quality of life is the only variable that can explain changes in the emotional impact of the pandemic in the study population. This finding should guide future psychoeducational interventions and services for adults.

## Introduction

Now that the COVID-19 pandemic has officially ended (WHO, 2023), it is wise to study the emotional impact it has had both on the population without psychiatric disorders and on people affected by a mental disorder. It is necessary to identify the risk factors and protective factors that influenced the emotional impact of this pandemic.

The consequences on mental health in the general population have been devastating, being more severe in those affected by a previous mental disorder, such as people with ASD^1–4^. In a previous study involving 53 adults who also participated in this research, this group was found to be particularly vulnerable, suffering deterioration in physical variables such as balance and severity of the core symptoms of the disorder^5^.

In addition, increased anxiety and stress in parents of children with autism, a greater need for support compared to the pre-pandemic period and difficulties in coping have been observed^6^. One of the most affected variables was emotional well-being, which was significantly influenced by the aggravation of behavioral problems and the frequency and usefulness of the support received^7^.

Other studies evidence the impact of the COVID-19 pandemic on people with ASD and its association with poorer well-being and greater depression, but not with suicide risk^8^.

This study aims to identify which have been the protective factors for adults with ASD and ID against the emotional impact of the pandemic, to develop intervention programs to prevent the development of negative symptomatology after periods of confinement, social isolation and health crises. For this purpose, the variables (social communication, emotional well-being and anxiety) that have previously been found to correlate with the emotional impact of the pandemic in this population^5,6,8^ were studied and their causal effect was analyzed.

These variables, in addition to having been affected by the pandemic, are also expected to be protective factors for adults with ASD and ID in future health crises, and would therefore form part of preventive intervention programs.

## Method

### Participants

The study has been conducted with a sample consisting of 60 subjects with ASD and ID (19 females and 41 males). Table 1 shows data on the degree of intellectual disability and chronological age, aged between 21 and 59 years old. As for the degree of intellectual disability, the IQ was taken into account, 0 being ‘light’ or ‘slight’ (IQ 56–75), 1 ‘moderate’ (IQ 46–55), 2 ‘severe’ (IQ 26–45) and 3 ‘profound’ (IQ 0–25)^9^.

**Table 1:** Subject demographics and baseline characteristics.

Sex	n	Male	Female	Total
		41	19	60
Chronological age	*M*	34.24	36.52	34.80
	*SD*	9.05	6.12	8.28
Grade of intellectual disability	*M*	1.86	2.09	1.94
	*SD*	1.14	0.53	0.96
0	%	18.2	0	11.8
1	%	18.2	27.3	20.6
2	%	27.3	30	38.2
3	%	36.4	18.2	29.4

Grade of intellectual disability; 0 = light or slight (IQ 56–75), 1 = moderate (IQ 46–55), 2 = serious (26–45), 3 = deep (0–25).

Participants attend to two day centers for adults with autism in Madrid and Vigo (Spain). All participants were clinically diagnosed with autism by a psychiatrist or clinical psychologist with several years of experience in the evaluation of these disorders. Both centers have been developing an adult education program for two decades with 110 people with autism. On this occasion, the development of the social communication in the population was encouraged, in addition to measuring the variables studied below (social competence, quality of life, impact of the pandemic, etc.). Out of the 110 people, 65 were participated in this project, for their familiarity with the use of the digital whiteboard and tablets (the didactic materials were implemented on these platforms) was prioritized. The only exclusion criteria in this initial group of 65 users was not having obtained informed consent (only two people were excluded for this reason) and being ill at the time of the study (3 people were excluded for this reason). The ethical committee of the Asociación Nuevo Horizonte reviewed and approved this study.

### Ethics declarations

Informed consent has been obtained from all subjects and/or their legal guardians. This research was conducted in accordance with the Declaration of Helsinki.

### Materials

The questionnaires used in this research were:

#### Vineland Adaptive Behavior Scales (VABS)^10^

VABS is an instrument that assesses social competence through 4 subscales: communication, daily living skills, socialization and motor skills. In this research, the total scores of 3 of the 4 subscales (Communication, Socialization and Daily Life Skills) were used. In this study, the raw scores in three subdomains were used (Communication, Socialization and Skills of Daily Living). "Communication” comprises three subdomains; receptivity, expressiveness and written communication, "Socialization” comprises three subdomains: interpersonal relationships, playtime, and coping skills. The "Daily Living Skills" comprises three subdomains: personal, domestic and community. Each item was scored from 0 to 2, 0 being that the activity or task is never performed, 1 that it is in progress and 2 that it has been acquired.

This instrument has been routinely used to assess the adaptive behavior of people with ASD^11–13^. The psychometric properties are sound, with high test–retest reliability (α = 0.98)^14^.

#### Personal Wellbeing Index (PWI)^15^

The Personal Wellbeing Index (PWI) of Cummins and Lau^15^ was applied. The PWI is a brief 7-item questionnaire (standard of living, personal health, achieving in life, personal relationships, safety, community-connectedness, and future security) that is very useful and valid for the evaluation of emotional well-being in people with autism and disability. Each item is assessed on a scale from 0 to 10, where 0 is "very dissatisfied" and 10 is "very satisfied”. These domains are summed up to yield an average score. This questionnaire has demonstrated good reliability and validity^16^.

#### COVID-19 Peritraumatic Distress Index (CPDI)^17^

The original questionnaire was published in Chinese by Qiu et al. and translated into English by the same authors^17^. It consists of four dimensions and 24 items in total. The objective is to measure the emotional impact (EI) caused by COVID; the frequency of anxiety, depression, specific phobias, cognitive change, avoidance and compulsive behaviour, physical symptoms and loss of social functioning in the past week, ranging from 0 to 100. A score between 28 and 51 indicates mild to moderate distress. A score ≥ 52 indicates severe distress. The Cronbach’s alpha of CPDI is 0.95 (p < 0.001)^17^.

#### Stress Survey Schedule for persons with autism and other developmental disabilities (SSS)^18^.

The SSS is a survey instrument to assess perceived stress reactions in the lives of people with autism and related disabilities. It consists of 49 items comprising eight stress scales: changes and threats, anticipation/uncertainty, unpleasant events, pleasant events, a sensory/personal contact, food-related activity, social interactions/environment, and ritual-related stress. Each item assesses the intensity of the anxiety displayed, where 1 is "none to mild", 2 "mild to moderate", 3 "moderate", 4 "moderate to severe" and 5 "severe".

The SSS was designed to be completed in three ways: (a) self-administered by the individual, (b) completed in the form of an interview, or (c) completed by the informant who knows the individual best. It is a valid tool for identifying which of the dimensions of stress are perceived as more or less stressful for subgroups of people with autism^19^.

### Procedure

The questionnaires were completed by three professionals trained in psychological diagnosis, familiar with the study population, but not with the research objectives. All questionnaires were completed in May 2022.

Each participant was given the necessary breaks, and some of the tasks were explained using communication systems used by each individual (pictograms, photographs, sign language, etc.). In all the questionnaires used (VABS, PWI, CPDI and SSS), the questions that could not be answered were completed in hetero-questionnaire mode. The raw scores of all questionnaires were used to perform the subsequent statistical analyses.

### Statistical analysis

Prior to analysis, data were examined. The residuals were normally distributed, and the assumption of homoscedasticity was met. None of the cases were identified as multivariate outliers, leaving all cases for the final analysis.

The correlations between all the variables included in the study were analyzed. Multiple linear regression analysis was used to analyze how the set of independent variables (area of communication writing, area of socialization leisure and well-being index) contribute to and explain the changes that occur in the dependent variable (emotional impact of the pandemic).

In the regression analysis, assumptions of collinearity were assessed through the evaluation of variance inflation factor (VIF) and tolerance statistics. Conservative cut-offs of VIF > 4 and tolerance < 0.20 were used, as described in Dormann et al.,^20^ and Lavery, Acharya, Sivo and Xu^21^.

Changes in multiple correlations squared (*R*^2^ change) were reported to demonstrate the amount of variance explained by each variable. All statistical analyses were computed using SPSS Statistical Software Package, version 27 for Mac.

## Results

A correlation analysis was performed with the purpose of confirming and analyzing the relationship between the variables included in the study.

The results of the correlation analysis (Table 2) show the existence of significant correlations between emotional impact of the pandemic and three independent variables: area of communication writing (*r* = −36, *p* < 0.01), area of socialization leisure (*r* = 0.272, *p* < 0.05) and well-being index (*r* = −0.327, *p* < 0.05).

**Table 2:** Pearson's bivariate correlation analysis between.

	1	2	3	4	5	6	7	8	9
1. VIN-RE	–								
2. VIN-EX	0.863**	–							
3. VIN-ES	0.702**	0.781**	–						
4. VIN-IN	0.869**	0.928**	0.823**	–					
5. VIN-OC	0.657**	0.728**	0.648**	0.791**	–				
6. VIN-BM	0.746**	−785**	0.735**	0.839**	0.772**	–			
7. PWI	0.225	0.142	0.133	0.258*	0.358**	0.253	–		
8. CPDI	0.156	0.232	0.367**	0.239	0.272*	0.212	-0.327*	–	
9. SSS	0.546**	0.441**	0.306*	0.465**	0.342**	0.359**	0.113	0.067	–

*VIN-RE* Vineland-acknowledgment, *VIN-EX* Vineland-expressiveness, *VIN-ES* Vineland-writing, *VIN-IN* Vineland-interactions, *VIN-OC* Vineland-leisure, *VIN-BM* Vineland-good manners, *PWI* wellbeing, *CPDI* emotional impact (EI) caused by COVID, *SSS* stress.

*p < 0.05, **p < 0.01, ***p < 0.001.

Subsequently, a multiple regression analysis was performed, in which the emotional impact of the pandemic was taken as the dependent variable. As independent variables area of communication writing, area of socialization leisure and well-being index were analyzed. The results of the analysis can be seen in Table 3.

**Table 3:** Hierarchical multiple regression analysis of the emotional impact of the pandemic.

Predictors	*R*^2^	*R*^2^ adjusted	Β	*B*	*F*	Tolerance	VIF
Model 1	0.315	0.277			*F*_(3,54)_ = 8.266***		
VIN-ES			0.118	0.244		0.571	1.75
VIN-OC			0.192	0.279		0.506	1.97
PWI			−0.280***	−0.460		0.854	1.17

*VIN-ES* Vineland-writing, *VIN-OC* Vineland-leisure, *PWI* wellbeing, *VIF* variance inflation factor.

*p < 0.05, **p < 0.01, ***p < 0.001.

The independent variables explain the 31% (adjusted *R*^2^) of the variance of the dependent variable (Table 3). The value of β indicated that well-being index (PWI) contributes significantly to improving the fit of the model (β = −0.280; *t* = −3.774; *p* < 0.001). The rest of the variables; communication writing (β = 0.118; t = 1.63; p > 0.05) and socialization leisure (β = 0.118; t = 1.63; p > 0.05) do not significantly contributed to improving the fit of the model.

## Discussion

The aim of this study was to show how social communication, communication social, emotional wellbeing and anxiety protect against emotional distress in adults with autism and intellectual disability during the covid-19 pandemic.

The results obtained in this study show a correlation between aspects of social communication such as writing, leisure, and the emotional impact of the pandemic. This supports some of the previous results that had pointed to a worsening of the severity of the core symptoms of autism^5^. However, it has not been possible to demonstrate that these variables are protective nor that they explain the increase in the negative impact of the pandemic, so this causal relationship cannot be established.

On the other hand, a significant correlation has been uncovered between quality of life or emotional well-being and the emotional impact of the pandemic. This coincides with previous results of several studies^7,8^ that proved the existence of this relationship. In addition, a causal relationship is evident in this study demonstrating that previous quality of life significantly explains the results achieved in the emotional impact of the pandemic.

These results can guide preventive interventions, giving special importance to adults with autism and ID to achieve an optimal perception of their standard of living, personal health, life achievements, personal relationships, safety, community connectedness, and security in the future.

### Limitations

Some significant limitations have been identified in this research, such as the absence of a control group to compare the results between different clinical profiles, in addition to the population with ASD and ID. Nor has it been possible to access a population that attends other types of more autonomous services, other than day and residential centers.

On the other hand, the latest version of some questionnaires has not been used, as in the case of the VABS^10^. This facilitates the comparison of results with previous studies using the same version, but entails other limitations that must be taken into account when interpreting the result.

Finally, to better analyze the causal relationship between quality of life and the emotional impact of the pandemic, the variables that are part of the quality-of-life construct must be analyzed separately and in more depth, since it is too complex and contemplates many areas of a person's daily life.

Despite these limits, this study has important strengths, such as the use of a standardized instruments, the inclusion of different regions of Spain, and the collection of information regarding the whole period of the Spanish lockdown. The use of this information can provide an important support for emotional distress prevention programs in the face of another epidemic. Future research should focus on both protective factors and risk factors that may affect the adult population with ASD and ID, thus providing more key variables to contribute to the promotion of well-being and quality of life for adults with ASD. In addition, it would be convenient to analyze these variables in other population profiles, including control groups in the design.

## Conclusion

The population with autism and ID has many difficulties in social relationships and changes in the environment and their routines^22^. In this study we have analyzed the variables that have correlated with the emotional impact of the pandemic (social communication, quality of life and anxiety) and subsequently performed a multiple regression analysis to find out to what extent these variables explain the changes in the emotional impact of the pandemic.

The results show that quality of life is the only variable that is able to explain changes in the emotional impact of the pandemic in the study population. This finding should guide future psychoeducational interventions and services for adults, since quality of life, composed of perception of their standard of living, personal health, life achievements, personal relationships, safety, community connectedness, and security in the future, is a protective factor in the face of health crises such as the one we have just experienced.

Therefore, these results support the creation of projects and services that improve inclusion in the community through leisure and employment, as well as the reinforcement of specialized health services that take into account the evolution, aging and comorbidities of ASD.

## Data Availability

The data sets used and/or analyzed during this study are available from the corresponding author upon reasonable request.
